# Breast cancer drugs: FDA approval, development time, efficacy, clinical benefits, innovation, trials, endpoints, quality of life, value, and price

**DOI:** 10.1007/s12282-024-01634-x

**Published:** 2024-09-25

**Authors:** Julia Caroline Michaeli, Thomas Michaeli, Dario Trapani, Sebastian Albers, Dominik Dannehl, Rachel Würstlein, Daniel Tobias Michaeli

**Affiliations:** 1 Department of Obstetrics and Gynaecology, LMU University Hospital, LMU Munich, Munich, Germany; 2 Department of Personalized Oncology, University Hospital Mannheim, Heidelberg University, Mannheim, Germany; 3 DKFZ-Hector Cancer Institute at the University Medical Center Mannheim, Mannheim, Germany; 4 Division of Personalized Medical Oncology, German Cancer Research Center (DKFZ), Heidelberg, Germany; 5 European Institute of Oncology, IRCCS, Milan, Italy; 6 Department of Oncology and Hematology, University of Milan, Milan, Italy; 7 Department of Orthopaedics and Sport Orthopaedics, School of Medicine, Klinikum Rechts Der Isar, Technical University of Munich, Munich, Germany; 8 Department of Women’s Health, Tuebingen University Hospital, Tübingen, Germany; 9 Department of Medical Oncology, National Center for Tumor Diseases, Heidelberg University Hospital, Im Neuenheimer Feld 460, 69120 Heidelberg, Germany

**Keywords:** Breast cancer, Early breast cancer, Cancer drugs, Clinical trials, Antibody-drug conjugates, FDA approval, Value-based pricing, Innovation, Biomarker, Antibody, HER2, Hormone receptor, BRCA, Quality of life, Overall survival, Progression-free survival, Tumor response, Objective response rate, ESMO-MCBS, Metastatic breast cancer

## Abstract

**Objective:**

This study analyzes the development, benefits, trial evidence, and price of new breast cancer drugs with US Food and Drug Administration (FDA) approval.

**Methods:**

We identified 26 drugs with 42 FDA-approved indications for early and metastatic breast cancer (2000–2023). Data were collected from FDA labels, clinicaltrials.gov, and Medicare and Medicaid. Overall survival (OS) and progression-free survival (PFS) hazard ratios (HRs) and tumor response’s relative risk (RR) alongside objective response rate (ORR) were meta-analyzed.

**Results:**

The median development time for breast cancer drugs was 7.8 years (95% CI 6.2–10.8). 26% of treatments were considered innovative (“first-in-indication”) with 88% acting via a targeted mechanism. 64% were small molecules, 19% antibodies, and 18% antibody-drug conjugates. 38% were approved for HR + and 31% for HER2 + breast cancer. 6 indications were for early and 36 for metastatic breast cancer. Indications utilized FDA’s special programs: orphan (2%), fast track (24%), accelerated approval (19%), priority review (74%), breakthrough therapy (44%). Approval was predominantly supported by phase 3 trials (88%) of randomized controlled design (66%), enrolling a median of 585 patients (IQR 417–752) at 181 centers (IQR 142–223) across 19 countries (IQR 17–20). New drugs’ HR were 0.78 for OS (95% CI 0.74–0.82) and 0.59 for PFS (95% CI 0.54–0.64) with a RR for tumor response of 1.61 (95% CI 1.46–1.76). Median improvements of OS were 2.8 months (IQR 1.8–5.8) and PFS were 4.4 months (IQR 2.2–7.1). In single-arm trials, the average ORR was 31% (95% CI 10–53). In meta-regressions, the correlation between OS/PFS was 0.34 (*p* = 0.031) and OS/response was 0.01 (*p* = 0.435). 60% of treatments had a ‘*high-value*’ ESMO-MCBS score with 14% demonstrating improvements in quality of life. The median price was $16,013 per month (95% CI 13,097–17,617). There was no association between prices and patient benefit. The median value per life year gained was $62,419 (IQR 25,840–86,062).

**Conclusions:**

Over the past two decades, the development of innovative and effective drugs transformed the treatment landscape for breast cancer patients. Yet, investigators and regulators must safeguard that highly-priced new drugs demonstrate improvements in patient-centered clinical endpoints: overall survival and quality of life.

**Supplementary Information:**

The online version contains supplementary material available at 10.1007/s12282-024-01634-x.

## Introduction

Breast cancer is a leading cause of morbidity, disability, and mortality in women around the world [1]. With over 2.3 million new cases and 685,000 deaths in 2020, breast cancer yielded a total loss of 20.6 million disability-adjusted life years (DALYs), equating to a 0.05% productivity loss in gross domestic product [2, 3]. In the US, these figures correspond to approximately 287,850 new cases and 43,250 deaths, resulting in USD 67 million of productivity loss in younger women, USD 246 million in midlife, and USD 66 million in older women in 2015 [4]. It is estimated that 5–10% of all patients with breast cancer are diagnosed with de novo metastatic disease, and a third of those diagnosed in early-stage will eventually experience metastatic recurrence [5]. The total costs associated with the management of patients with metastatic breast cancer are estimated to be USD 63.4 billion in 2015 and are expected to rise to USD 152.4 billion with the current trend [6]. De facto, such an estimate is still very conservative, because cost implications for the treatment of breast cancer, with emerging new active drugs prolonging the disease control and adding on new options throughout the therapeutic journey of patients, come with increasingly higher prices.

However, not all the new medicines approved by the US Food and Drug Administration (FDA) are equally innovative and effective. Only a few FDA-approved medicines improve relevant patient-centered outcomes, e.g., overall survival (OS) and quality of life (QoL) [7]. Across 124 new cancer drugs with FDA approval across 374 indications (2003–2021), the median OS improvement was 2.8 months, with a median progression-free survival (PFS) improvement of 3.3 months [8]. Improvements in QoL are rarely demonstrated, and more commonly QoL data are just not reported, albeit more consistently collected in recent years [9, 10].

Despite these disputes surrounding cancer drugs’ clinical benefits, their launch and post-launch prices have been steadily rising in the US [11, 12]. Among injectable cancer drugs, median monthly prices of USD 2,800 were reported in 2005, surging to USD 15,000 in 2023. Of note, a large study of 145 cancer drugs with 337 indications found that drug prices are (at best) only poorly correlated with the clinical benefits (OS, PFS, tumor response) they offer to patients [11]. Similarly, another study found no correlation between the European Society for Medical Oncology Magnitude of Clinical Benefit Scale (ESMO-MCBS) and monthly treatment costs in the US, England, Germany, France, and Switzerland [13].

The space of breast cancer is particularly brisk in terms of ongoing clinical trials to meet the significant burden of patients, bringing unique challenges and clinical needs. However, no study dedicated to breast cancer has so far evaluated the clinical evidence supporting and patient benefits provided by new FDA drug approvals and price implications. As such, we investigated this area, to understand if general trends in oncology are also applicable to breast cancer, or if the situation for this tumor differs. This study analyzes the development, benefit, trial evidence, price, and cost-effectiveness of novel breast cancer drugs with FDA approval.

## Data and methods

### Sample identification

We accessed the Drugs@FDA database to identify all new drugs, including New Drug Applications and Biologic License Applications, with FDA approval between 1 January 2000 and 30 June 2023. The sample was then restricted to include only anti-cancer drugs, excluding non-oncology, supportive care, and diagnostic agents, while including gene and cell therapies. For each drug, we identified all anti-cancer indications approved until 30 June 2023. The sample was then restricted to only include indications approved for the treatment of early (perioperative) and metastatic breast cancer.

### Data collection

We collected data on the FDA approval, clinical trial evidence, cancer epidemiology, and price for each cancer drug and indication from publicly available sources (Table e1). For each indication, FDA labels were accessed to collect data on the drug, indication, and clinical trial characteristics. The first reviewer (D.T.M.) independently retrieved data from FDA labels, which was then cross-checked by the second reviewer (T.M.) with data found on clinicaltrials.gov and associated peer-reviewed publications. Disagreements were resolved in consensus or by consulting an experienced oncologist. Full details of the data extraction method have previously been described elsewhere [8, 14].

#### Clinical development times

For all original breast cancer indication approvals, we collected data on the date the initial new drug application (IND) became effective and the FDA approval date from FDA documents or the US Patent and Trademark Office (USPTO).

#### Drug characteristics

Drugs were categorized by their mechanism of action (cytotoxic chemotherapy vs. targeted agent vs. immune regulator) and product type (small molecule vs. antibody vs. antibody-drug conjugate).

#### Special FDA designations and review

For each indication, we collected data on the use of the following special FDA designations and review programs from official FDA websites: orphan designation, fast track, accelerated approval, priority review, and breakthrough therapy designation [15].

#### Indication characteristics

Indications were then categorized by treatment type (monotherapy vs. combination therapy), biomarker status (hormone receptors [HRs] positive vs. human epidermal growth factor receptor 2 [HER2] positive vs. other vs. none), and line of therapy (first-line vs. second-line vs. ≥ third-line). Clinical novelty and innovativeness were determined on an indication level based on a previously reported definition of indication novelty [16]: We differentiated drugs for new indications (first-in-indication), drugs for known indications with a major benefit as exhibited by FDA priority review (advance-in-indication), and drugs for known indications without FDA priority review (addition-to-indication).

#### Pivotal clinical trials

Each indication’s pivotal trial was characterized by the number of enrolled patients, phase (phase 2 vs. phase 3), design (randomized controlled trial [RCT] vs. single-arm trial), blinding (open-label vs. double-blind), number of arms, direct comparator (active [anti-cancer drug] vs. inactive [placebo or no treatment]), primary endpoint (OS vs. PFS vs. tumor response), randomization ratio (equal vs. skewed), and crossover (allowed vs. not allowed vs. not specified). For RCTs, we extract hazard ratios (HRs) for OS and/or PFS and/or the relative risk (RR) of tumor response with 95% confidence intervals (CI). The number of subjects and events was noted for the control and intervention arms. For single-arm trials, we calculated the objective response rate (ORR) based on the number of responders and subjects. Median improvements in OS, PFS, and duration of tumor response with interquartile ranges (IQR) were calculated as the difference between median survival in the treatment and control arm.

#### Clinical benefit and quality of life

Each indication's benefit was determined using the ESMO-MCBS in 2023. In the metastatic setting, an ESMO-MCBS of 4 to 5 was judged as “high-value”, while an ESMO-MCBS of 1 to 3 was judged as “low-value”. In the curative setting, an ESMO-MCBS of “A” and “B” was judged as “high-value”, while an ESMO-MCBS of “C” was judged “low-value”. Furthermore, we collected data on indications’ improvement in QoL listed by the ESMO-MCBS.

#### Drug prices

Drug prices were retrieved in August 2023 from the Centers for Medicare & Medicaid Services (CMS) and Medicare’s plan finder tool for patients covered under Medicare Part B and D, respectively. Coherent with previous studies [17–21], monthly treatment costs were estimated for an average adult with a body surface area of 1.7m^2^ weighing 70 kg based on the dosing regimen in the FDA label. Full details of the drug price calculation have previously been described elsewhere [11, 12].

### Statistical analysis

Breast cancer treatments were compared across product types regarding their clinical development time, drug characteristics, special FDA designations, indication characteristics, pivotal clinical trials, efficacy, price, and cost-effectiveness.

First, clinical development times, calculated as the difference between IND to NDA/BLA approval for original indications, were compared using Kaplan–Meier survival curves, log-rank tests, and a Cox proportional hazard model. Second, the distribution of categorical variables describing new breast cancer treatments’ drug, special FDA designations, indication, and pivotal clinical trial characteristics was compared with Fisher’s exact tests. Medians were compared with Kruskal–Wallis test. Third, a series of random-effects meta-analyses were conducted for clinical trials with available outcome data. HRs for OS and PFS were meta-analyzed in all RCTs. RRs for tumor response were meta-analyzed in all RCTs. ORRs were meta-analyzed in all single-arm trials. Differences between indications’ HRs, RRs, and ORRs were compared with Cochran’s Q test. Fourth, we evaluated the association between OS and PFS as well as OS and tumor response outcomes using meta-regression and weighted least squares (WLS) regression analyses for breast cancer drugs in the metastatic setting. Fifth, mean monthly drug prices were compared in January 2023 and correlated to OS and PFS outcomes. Finally, we calculated the incremental cost per life-year gained for OS and PFS.

Analyses were stratified by product type (small molecule vs. antibody vs. antibody–drug conjugate), biomarker status (HR-positive vs. HER2-positive vs. other vs. none), treatment setting (early breast cancer vs. metastatic breast cancer), and number of special FDA designations.

Data were stored in Microsoft Excel (Microsoft Corp) and analyzed with Stata software, version 14.2 (StataCorp LLC). Two-tailed *p* values below 0.05 were considered significant. This study followed the Strengthening the Reporting of Observational Studies in Epidemiology (STROBE) reporting guidelines where applicable [22].

## Results

### Sample overview

The FDA approved 26 unique cancer drugs for the treatment of breast cancer across 42 indications between 2000 and 2023. Of these 42 indications for breast cancer, 27 (64%) were small molecules, 8 (19%) antibodies, and 7 (18%) antibody–drug conjugates (Table 1). 6 indications were for early breast cancer and 36 for metastatic breast cancer.

**Table 1:** Characteristics of drug indications with FDA approval for breast cancer from 2000 to 2023

Variables	No. (%)
**Drug Characteristics**	
Indication novelty	
Addition-to-indication	11 (26)
Advance-in-indication	20 (48)
First-in-indication	11 (26)
Mechanism of Action	
Cytotoxic Chemotherapy	2 (5)
Targeted Agents	37 (88)
Immune Regulators	3 (7)
**FDA Approval**	
Orphan Drug Designation	1 (2)
Fast Track Designation	10 (24)
Accelerated Approval	8 (19)
Priority Review	31 (74)
Breakthrough Therapy Designation ^a^	16 (38)
**Indication Characteristics**	
Indication Approval Type	
Original indication approval	25 (60)
Supplemental indication approval	17 (40)
Treatment Type	
Combination	27 (64)
Monotherapy	15 (36)
Line of Therapy	
First-line	18 (43)
Second-line	18 (43)
≥ Third-line	6 (14)
**Pivotal Clinical Trial Characteristics**	
Enrolled patients, median (IQR)	585 (417–752)
Accrual rate, median (IQR) ^b^	34 (17–49)
Clinical trial sites, median (IQR)	181 (142–223)
Participating countries, median (IQR)	20 (17–19)
Trial Phase	
Phase 2	5 (12)
Phase 3	37 (88)
Trial Design	
Single-arm	2 (5)
Randomized-controlled	40 (95)
Type of Blinding	
Open-Label	21 (50)
Double-Blind	21 (50)
Primary endpoint	
Overall Survival	3 (7)
Progression-Free Survival	29 (69)
Tumor Response	4 (10)
Total Concurrent RCTs, No	
Direct Comparator	
Inactive (Placebo or No Treatment)	29 (69)
Active (Cancer Drug)	13 (31)
Randomization ratio ^c^	
Equal	24 (60)
Skewed	16 (40)
Crossover	
Not specified	23 (58)
Allowed	1 (3)
Not allowed	16 (40)
**Total No. of Indications**	42 (100)

This table presents the drug, indication, special FDA designations and review programs, and pivotal clinical trial characteristics for indications with FDA approval for breast cancer. Approvals were compared across product types for small molecules, antibodies, and antibody–drug conjugates

*FDA* US Food and Drug Administration

^a^The comparison of the breakthrough therapy designation only includes indications approved after 2012, given that the program was initiated in 2012

^b^The accrual rate was calculated as the number of patients enrolled in the clinical trial per month

^c^Randomization ratios were stratified by the allocation proportion to the treatment and control arm in equal (e.g., 1:1, 1:1:1) and skewed (e.g., 2:1, 3:1, 2:1:1)

### Clinical development times

**Fig. 1:**
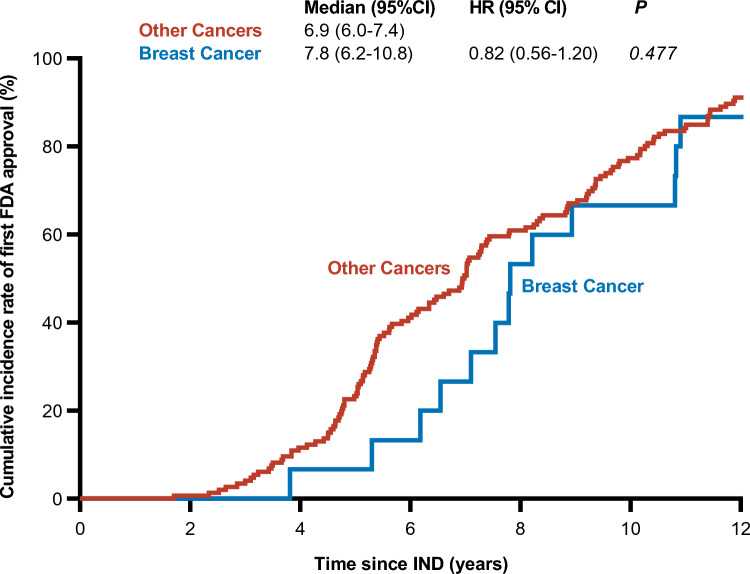
Time from IND to first FDA approval for breast cancer drugs. The graph illustrates the cumulative incidence of first FDA approval for drugs treating breast cancer (blue) and other cancers (red) in their first approved indication. *FDA* US Food and Drug Administration, *HR* hazard ratio, *IND* investigational new drug application

The median development time, defined as the time from IND to FDA approval, was 7.8 years (95% CI 6.2–10.8) for drugs treating breast cancer and 6.9 (95% CI 6.0–7.4) for drugs treating other cancers. However, this difference was not significant in the Cox proportional hazard model (0.82, 95% CI 0.56–1.20, *p* = 0.477) (Fig. 1).

### Drug characteristics

Among the 42 breast cancer indications, 11 (26%) were first-in-indication, 20 (48%) were advance-in-indication, and 11 (26%) were addition-to-indication. Small molecules, antibodies, and ADCs did not significantly differ in their novelty although there was a higher percentage of first-in-indication treatments among ADCs (22% vs. 25% vs. 43%, *p* = 0.772). The majority of indications acted via a targeted (88%) mechanism of action than immune-regulating (7%) or cytotoxic (5%) mechanisms (Table 1).

### Special FDA designations and review

Out of all the 42 breast cancer indications, 74% received priority review, 44% breakthrough therapy designation, 24% fast track, and 19% received accelerated approval. Accelerated approval was more frequently granted to antibodies and ADCs than small molecules (50% vs. 29% vs. 7%, *p* = 0.012) (Table e2). Surprisingly, one indication received an orphan designation, granted to tucatinib “in combination with trastuzumab and capecitabine for the treatment of adult patients with advanced unresectable or metastatic HER2-positive breast cancer, including patients with brain metastases, who have received one or more prior anti-HER2-based regimens in the metastatic setting” based on the HER2CLIMB trial [23]. Orphan designations for subsets of common diseases are referred to as “common orphans” and have previously been reported for lung and skin cancer [14]. Importantly, they fall outside the definition and scope of other regulatory agencies’ definition of an orphan disease, e.g. the European Medicines Agency.

Table e3 demonstrates that more special designations were associated with smaller pivotal trials that were less likely to be of randomized controlled phase 3 design. More special FDA designations were associated with a greater efficacy. Benefits for OS (HR: 0.88 vs. 0.81 vs. 0.73 vs. 0.76 vs. 0.66, *p* = 0.035), PFS (HR: 0.78 vs. 0.61 vs. 0.55 vs. 0.55 vs. 0.54, *p* = 0.012), and tumor response (RR: 1.17 vs. 1.98 vs. 1.57 vs. 2.25 vs. 1.78, *p* < 0.001) improved with more special designations. In single-arm trials, the ORR was 12%, 26%, and 60% for indications with one, three and four special designations (*p* < 0.001). There was no significant association between the number of special designations and drug prices.

### Indication characteristics

New breast cancer treatments were predominantly original indication approvals (60%) for first-line (43%) or second-line (42%) combination treatments (64%). The majority of indications were approved with a biomarker (86%). The most frequently used biomarkers were HER2 and HR (Table 2).

**Table 2:** Biomarkers supporting the FDA approval of breast cancer drugs

Biomarker	No	(%)
HER2-positive	13	(31)
HR-positive	16	(38)
PD-L1	2	(5)
gBRCA	2	(5)
HER2 low	1	(2)
PIK3CA-positive	1	(2)
ESR1-positive	1	(2)
None	6	(14)
**Total**	**42**	**(100)**

*ESR1* estrogen receptor 1, *FDA* US Food and Drug Administration, *gBRCA* germline breast cancer gene, *HER2* human epidermal growth factor receptor 2, *HR* hormone receptor, *PD-L1* programmed death ligand 1, *PIK3CA* phosphatidylinositol-4,5-bisphosphate 3-kinase catalytic subunit alpha

### Pivotal clinical trials

The FDA approval of breast cancer indications was supported by pivotal clinical trials with a median of 585 patients (IQR: 417–752) with an accrual rate of 34 patients per month (IQR: 17–49). Trials were conducted at a median of 181 sites (IQR: 142–223) across 19 countries (IQR: 17–20). An equal share of trials was double-blinded and open-label. Trials were predominantly phase 3 (88%) concurrent RCTs (95%) assessing PFS (69%) rather than OS (7%) as the primary endpoint (Table 1). The direct comparator was inactive (e.g. placebo or no treatment) for two-third of RCTs, with merely 33% comparing the new drug directly to active comparator (e.g., anti-cancer agent).

ADCs were more frequently supported by single-arm trials (29% vs. 0% vs. 0%, *p* = 0.024) of open-label blinding (100% vs. 38% vs. 41%, *p* = 0.012) than antibodies and small molecules (Table e2). RCTs supporting ADCs were more frequently assessing tumor response as the primary endpoint (29% vs. 25% vs. 0%, *p* = 0.012) and comparing the new drug to an inactive rather than an active comparator (100% vs. 13% vs. 26%, *p* = 0.003) than antibodies and small molecules. Stratification of the descriptive statistics across biomarkers is presented in Table e4.

Notably, clinical trials evaluating new drugs for early breast cancer were supported by more enrolled patients than those for metastatic breast cancer (median: 1745 vs. 540, *p* = 0.007). Early breast cancer trials showed a tendency to be conducted at more trial sites (median: 384 vs. 174, *p* = 0.057) and in more countries (median: 33 vs. 19, *p* = 0.062) with consequently significantly greater patient enrollment per month (median: 85 vs. 31, p = 0.011). Stratification of the descriptive statistics across treatment setting is presented in Table e5.

### Efficacy, clinical benefit, and quality of life

**Fig. 2:**
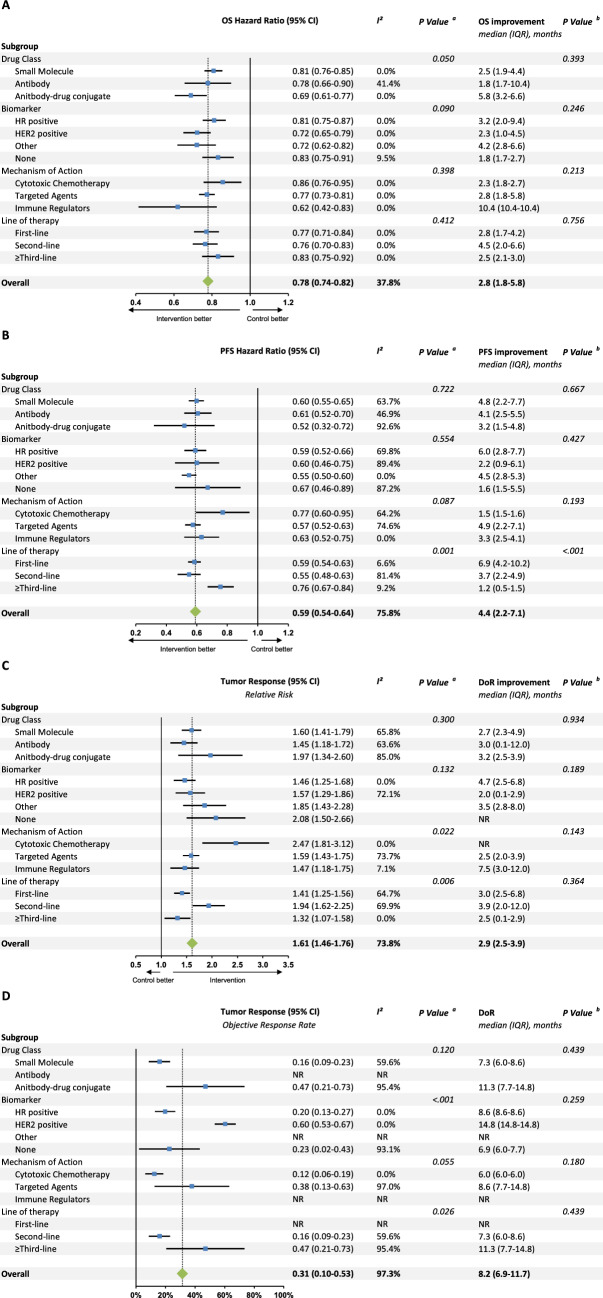
Meta-analysis of overall survival, progression-free survival, and tumor response supporting the FDA approval of breast cancer drugs. For randomized controlled trials, overall survival (graph **A**), progression-free survival (graph **B**), and tumor response rates (graph **C**) are meta-analyzed using random-effects models. For single-arm studies, the overall response rate is meta-analyzed (graph **D**). A continuity adjustment of 0.5 for control arms with 0 responders was applied for tumor responses. ^a^*P*-values calculated based on Cochran’s Q test for subgroup differences. ^b^*P*-values calculated based on Kruskal–Wallis tests. *DoR* duration of response, *FDA* US Food and Drug Administration, *IQR* interquartile range, *NR* not reported, *OS* overall survival, *PFS* progression-free survival

Among RCTs with available data, new breast cancer indications significantly reduced the likelihood of death with an HR of 0.78 (95% CI 0.74–0.82) and were associated with a median 2.8 months (IQR: 1.8–5.8) longer OS than control (Fig. 2). For PFS, the average HR was 0.59 (95% CI 0.54–0.64) with a median PFS improvement of 4.4 months (IQR: 2.2–7.1) compared to control. For tumor response, the RR was 1.61 (95% CI 1.46–1.76) with a median duration of response of 2.9 months (IQR: 2.5–3.9) relative to control. In single-arm trials with available data, the average ORR was 31% (95% CI 10–53) with a median duration of response of 8.2 months (IQR: 6.9–11.7). ADCs were associated with a greater OS benefit relative to antibodies and small molecules in terms of HR (0.69 vs. 0.78 vs. 0.81, *p* = 0.050). There was no difference in PFS (HR: 0.80 vs. 0.78, *p* = 0.836) and tumor response (RR: 1.83 vs. 1.61, *p* = 0.600) for drugs treating early and metastatic breast cancer. 60% of treatments had a high-value ESMO-MCBS score with 14% demonstrating improvements in QoL and 41% in OS.

### Correlation between OS, PFS, and tumor response

**Fig. 3:**
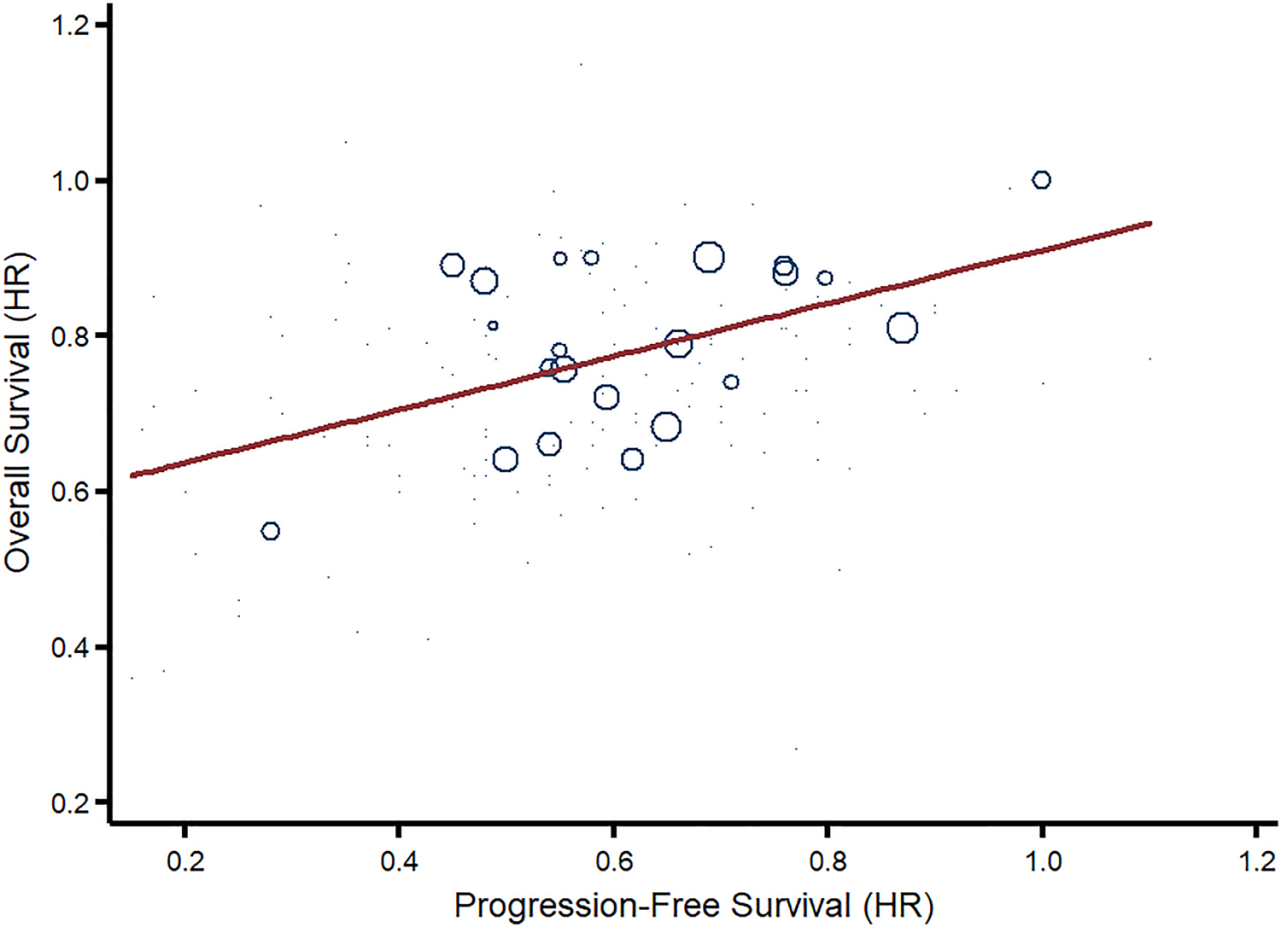
Association between overall survival and progression-free survival benefit in clinical trials supporting the FDA approval of breast cancer drugs. In the graph, each indication’s overall survival hazard ratio (*y*-axis) is mapped against its progression-free survival hazard ratio (*x*-axis). Within the graphs, the red line presents fitted treatment outcomes of the random-effects meta-regression. Circle sizes are subject to the precision of each treatment outcome, the inverse of their within-study variance. The analysis only includes treatment outcomes from drug indications with available data from randomized controlled trials. In the meta-regression, a correlation coefficient of 0.34 (95% CI 0.03 to 0.65, *p* = 0.031) was measured with an adjusted *R*^2^ of 78.4%. *FDA* US Food and Drug Administration, *HR* hazard ratio

We conducted a meta-regression of PFS hazard ratios on OS hazard ratios to evaluate the association between the surrogate endpoint PFS and the clinical endpoint OS. A correlation coefficient of 0.34 (95% CI 0.03 to 0.65, *p* = 0.031) was measured between OS and PFS HRs with an adjusted *R*^2^ of 78.4% (Fig. 3). We conducted a WLS regression analysis, weighted by the number of patients enrolled in the pivotal clinical trial, to evaluate the association between median improvements in PFS and OS. A correlation coefficient of 0.20 (95% CI − 37 to 0.78, *p* = 0.460) was measured between OS and PFS improvements.

Within conducted meta-regressions, there was no significant association between OS HR and tumor response RR (0.01, 95% CI − 0.02 to 0.05, *p* = 0.435) and PFS HR and tumor response RRs (− 0.03, 95% CI − 0.07 to 0.01, *p* = 0.100). Within conducted WLS regressions, there was a significant association between OS and duration of response improvements (1.24, 95% CI 0.16 to 2.32, *p* = 0.032) and PFS and duration of response improvements (0.26, 95% CI 0.13 to 0.40, *p* = 0.003).

### Drug prices

**Fig. 4:**
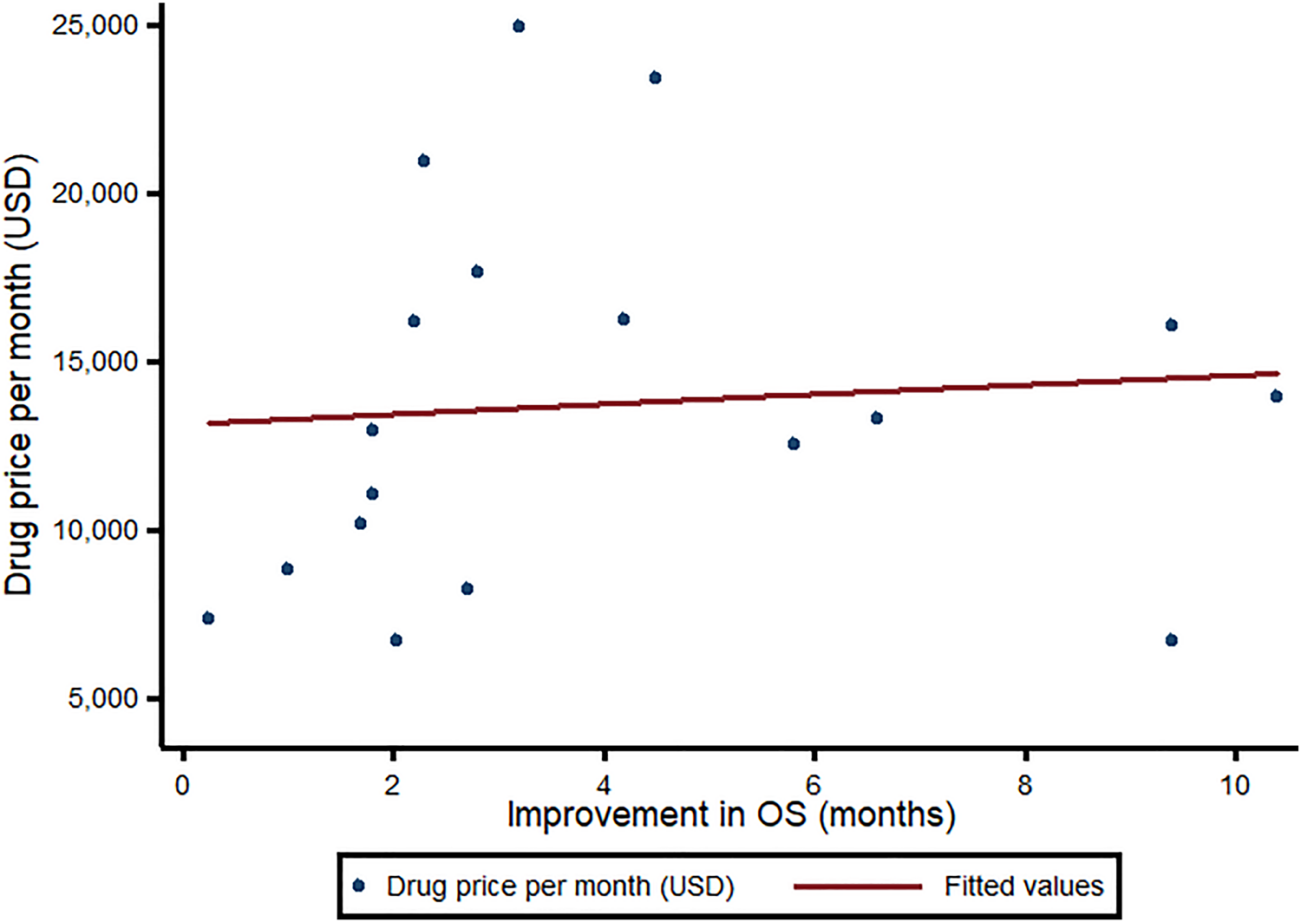
Association between monthly prices and improvement in OS for breast cancer drugs. This graph maps the monthly treatment costs of breast cancer indications (*y*-axis) against the improvement in OS. The improvement in OS was calculated as the different between the experimental and control arm in pivotal randomized-controlled trial. All prices are in 2023 USD. *FDA* US Food and Drug Administration, *OS* overall survival

In 2023, breast cancer drugs cost an average of $16,013 (95% CI 13,097 to 17,617) (Table e6). There was no association between monthly drug prices and the median improvement in OS (*ß* = 0.83, 95% CI − 3.11 to 4.77, *p* = 0.661) or PFS (*ß* = − 0.54, 95% CI − 4.47 to 3.39, *p* = 0.780) (Fig. 4). Accordingly, there was no significant difference in median prices for drugs that did and did not demonstrate a benefit in OS (USD 16,079 vs. 16,013, *p* = 0.891), QoL (USD 16,234 vs. 16,013, *p* = 0.319), or either (USD 16,146 vs. 16,013, *p* = 0.729). Coherently, there was no significant difference in prices for indications considered high-value vs. low-value based on the ESMO-MCBS (USD 16,013 vs. 16,243, *p* = 0.299). The median value per life year gained was $62,419 (IQR 25,840–86,062) for OS and $48,053 (IQR 27,265–77,894) for PFS.

## Discussion

The development of new innovative drugs has transformed the treatment landscape for patients with breast cancer. Pharmaceutical companies have dedicated substantial R&D resources toward breast cancer, as nearly 20% of new cancer drugs received approval for breast cancer over the past two decades. In this study, we shed light on the unique difficulties of the development, clinical evaluation, regulatory approval, and pricing of these new treatments.

For breast cancer, new drug approvals are typically supported by standard FDA approval rather than accelerated approval. We found that less than 20% of all new indications received accelerated approval, which is much lower than observed for other cancer entities [15]. Of note, more than two-third of the FDA accelerated approvals are for oncology indications, as reported in a cross-section investigation of approvals since the 2000s, corresponding to nearly half of all new approvals [24]. Breast cancer appears to be based on mostly randomized controlled phase 3 trials. Early-phase trials leading *tout droit* to drug approval are usually less common in breast cancer than other tumors. Yet, patient-centric clinical endpoints, such as OS and QoL, are typically not prioritized, consistent with the general trends in drug development, and are used as primary endpoints only in less than 20% of all trials leading to approval [25]. Measuring surrogate endpoints, such as PFS and tumor response, rather than OS, leads to faster patient access to novel drugs by reducing clinical development times by 11 months and 18 months, respectively [26]. However, the correlation of surrogate endpoints with patient-centered clinical endpoints remains weak. [27, 28] In our study, we confirm that this finding also applies to breast cancer, as we only found a weak correlation between OS and PFS. Therefore, a benefit in PFS, disease-free survival, recurrence-free survival, or tumor response may not necessarily result an improvement of OS or QoL. Recent trial results with CDK4/6 inhibitors highlight that PFS may not be a surrogate endpoint to predict OS or even QoL outcomes [29]. Regulators established multiple expedited approval pathways to provide patients with swift access to new, promising treatments. Similar to prior studies, greater efficacy estimates were measured for indications with multiple special FDA designations. Yet, they were also more frequently supported by smaller non-robust trials, which could overestimate efficacy outcomes, particularly for surrogate endpoints [30]. Therefore, regulators ought to ensure that confirmatory trials are conducted with clinically relevant endpoints to safeguard that these new treatments uphold their optimistic promise. Moreover, RCTs must transparently report post-progression treatment and cross-over to adequately compare the treatment sequence [31, 32]. Although drug sponsors frequently argue that the option to cross-over from the control to the treatment arm entices patient enrollement, to date there is no evidence supporting this statement [33]. Further,Concerns were raised that new drugs should be tested in the first- vs. second-line setting. However, only a few patients received the investigational compound after progressing on the treatment in the control arm. Notably, in the MONALEESA-7 trial, only 74% of patients in the ribociclib arm received any therapy, and only 69% of patients in the control arm [34].

An important path to enrich the population of patients deriving the greatest benefits from new cancer drugs is by enhancing their selection in biomarker-based clinical trials. Biomarkers are key for treatment selection in breast cancer. Specifically, breast cancer treatment is oriented by the basic biological characteristics, such as HRs expression and HER2 status. We show that over 90% of drug development in breast oncology is based on a biomarker. Furthermore, our study suggests that biomarker-driven drug development in breast cancer could portend better survival and treatment response outcomes. However, innovative biomarkers beyond HRs and HER2 are still limited. Innovative biomarkers include *PIK3CA*, *AKT* and related pathways, *ESR1*, germinal *BRCA,* and *PD-L1*.

While the benefit of breast cancer indications was heterogeneous in terms of hazard ratios and absolute gain in survival, there was no clear correlation with their prices in 2023. This is an unfortunate case in oncology. Value for money is commonly not recapitulated by how much resources are invested, with broad hype for innovation and new cancer indications *tout court*, but poor value-based attitudes and priority-setting [35, 36]. A lack of correlation between the price of cancer drugs at launch, survival improvement, and therapeutic value has been reported [11, 12]. Coherently, this study confirms this trend for breast cancer approvals over the past two decades.

Our cross-sectional analysis of breast cancer indications elucidated some key differences in drug approval in this setting. Breast cancer is still poorly permeated by non-controlled studies leading to approvals, for the availability of well-consolidated, multiple lines of therapy, based on comparative clinical trials [37, 38]. Arguably, the basic problem is still in the choice of endpoints to demonstrate that new drugs are better than existing treatment standards, and a major issue with their validation before the full implementation in the practice of clinical trials. While PFS can be measured in a shorter interval of time and is not affected by previous and later lines of therapy, therapeutic success cannot be assumed or proclaimed unless patient-centric metrics are shown to be improved, particularly “how much” an individual lives, and how the patients “feel during the days”. This is a basilar requirement, as recently emphasized by the Common Sense Oncology movement [39]. As such, we believe that the choice of co-primary or dual endpoints can portend multiple benefits. First, it could measure interim cancer-related metrics of response; then, it can measure therapeutic success based on gold-standard metrics, albeit requiring longer follow-up to be mature. A trigger to follow-up patients for longer intervals, for example through protocol-specified long-term analysis, can also improve knowledge on the value of new endpoints, and give more information on the validity of surrogate endpoints. Such monitoring should be required by regulatory agencies and could benefit from transparent, open, or for-purpose access to clinical trial databases from relevant stakeholders. Sticking to the paradigm of comparative studies, against the best standard of care, and validating new treatments in the context of biomarker-driven phase 3 trials seems to be the key to success, and so far better-pursued in the case of breast cancer. In our analysis, biomarker-based studies portend better outcomes. Leaving ample uncertainties in who derives benefit, and to what extent, can only generate uncertain results, and jeopardize the definition of optimal cancer care to shape the best treatment sequence. Also, it can expose patients less likely to derive a benefit from toxic treatments, resulting in possible clinical detriment.

## Conclusion

In conclusion, we demonstrate that there are key features in breast cancer drug development that should be preserved and optimized. Ideally, clinical trials must measure and demonstrate an improvement in OS and QoL. At the same time, policy-makers must stipulate pharmaceutical companies to develop new breast cancer drugs with innovative product types, e.g. ADCs, innovative mechanisms of action, and validated biomarkers. Investigators and regulators should strive to include patient-centered clinical endpoints, randomization, and active comparators into the design of pivotal clinical trials to deliver relevant outcomes. Finally, our study highlights the misalignment between new cancer drugs' value and price. Value frameworks, such as the ESMO-MCBS, could guide price negotiations and help identify high-value treatments that should be swiftly made available to breast cancer patients across the globe.

## Supplementary Information

Below is the link to the electronic supplementary material.

Supplementary file1 (PDF 701 KB)

## Data Availability

All data used in this study were in the public domain. All data relevant to the study are included in the article or uploaded as supplementary information.

## Declarations

### Provenance and peer review

Not commissioned; externally peer-reviewed.

### Patient and Public Involvement

No patients were involved in the conduct of this study.
