# Supplementary material for: Breast cancer drugs: FDA approval, development time, efficacy, clinical benefits, innovation, trials, endpoints, quality of life, value, and price
Source: Breast Cancer. 2024 Sep 25;31(6):1144–55. doi: 10.1007/s12282-024-01634-x (PMC11489271; doi:10.1007/s12282-024-01634-x)
Supplement: Supplementary file 1 — Supplementary file1 (PDF 701 KB) [file 12282_2024_1634_MOESM1_ESM.pdf]

Supplementary Online Content

J.C. Michaeli, T. Michaeli, D. Trapani, S. Albers, D. Dannehl, R. Würstlein, D.T. Michaeli (2024). Breast cancer drugs: FDA approval, development time, efficacy, clinical benefits, innovation, trials, endpoints, quality of life, value, and price. *Breast Cancer*. doi:10.1007/s12282-024-01634-x

Table e1. Data sources

Table e2. Stratification by product type: small molecules vs. antibodies vs. antibody-drug conjugates

Table e3. Stratification by number of special designations

Table e4. Stratification by biomarkers: HR positive vs. HER2 positive vs. other vs. none

Table e5. Stratification by treatment setting: early vs. metastatic breast cancer

Table e6. Median monthly price of breast cancer drugs

This supplementary material has been provided by the authors to give readers additional information about their work.

Source	Variable	Website
FDA label	Indication	https://www.accessdata.fda.gov/scripts/cder/daf/
	Indication approval date	
	Treatment type	
	Biomarker	
	Line of therapy	
	Drug dosing regimen	
FDA label and clinicaltrials .gov	Clinical trial enrolled patients	https://www.accessdata.fda.gov/scripts/cder/daf/ https://clinicaltrials.gov/
	Clinical trial design	
	Clinical trial phase [a]	
	Clinical trial blinding	
	Clinical trial primary endpoint	
	Clinical trial comparator	
	Clinical trial randomization ratio	
	Clinical trial endpoint outcome	
WHO	Drug innovation and indication novelty [b]	https://www.whocc.no/atc_ddd_index/
Drug Bank	Mechanism of action	https://go.drugbank.com/
	Product type	
Medicare [c]	Prices Medicare Part B	https://www.cms.gov/Medicare/Medicare-Fee-for-Service-Part-B-Drugs/McrPartBDrugAvgSalesPrice
	Prices Medicare Part D	https://www.medicare.gov/plan-compare/#/?lang=en&year=2022
FDA label / Federal register	IND date [d]	https://www.federalregister.gov/
	FDA approval date	https://www.accessdata.fda.gov/scripts/cder/daf/
FDA	Orphan Designation	https://www.accessdata.fda.gov/scripts/opdlisting/oopd/
	Fast Track	https://www.fda.gov/drugs/nda-and-bla-approvals/fast-track-approvals
	Accelerated Approval	https://www.fda.gov/drugs/nda-and-bla-approvals/accelerated-approvals
	Priority Review	https://www.fda.gov/drugs/nda-and-bla-approvals/priority-nda-and-bla-approvals
	Breakthrough Therapy	https://www.accessdata.fda.gov/scripts/cder/daf/

Table e1. Data sources

[a] Combined phase 1/2 trials were classified as phase 2, combined phase 2/3 as phase 3.
[b] Drugs were categorized as "first-in-class", "advance-in-class", and "addition-to-class" according to Lanthier et al.'s methodology of defining drug innovation. Further, indication novelty was accordingly assessed to define indications as "first-in-indication", "advance-in-indication", and "addition-to-indication".
[c] For drugs without available data from Medicare data sources, prices were retrieved from the drug abacus (https://www.drugpricinglab.org/).
[d] The date when the IND became effective was primarily obtained from "Determination of Regulatory Review Period for Purposes of Patent Extension" documents submitted by the FDA to the US Patent and Trademark Office USPTO. For drugs without these documents, the date when the IND became effective was determined 30 days after the IND was submitted to the FDA as disclosed in FDA review documents.

Abbreviations: CPI, consumer price index; DALY, disability-adjusted life years; FDA, US Food and Drug Administration; IND, investigational new drug application; USPTO, US Patent and Trademark Office; WHO, World Health Organization; YLD, year lived with disability; YLL, years of life lost.

No. (%)					
	FDA approvals for breast cancer				Overall
Variables	Small Molecule	Antibody	Antibody-Drug Conjugate	*P Value* [a]	
**Drug Characteristics**					
Indication novelty				*0.772*	
Addition-to-indication	7 (26)	3 (38)	1 (14)		11 (26)
Advance-in-indication	14 (52)	3 (38)	3 (43)		20 (48)
First-in-indication	6 (22)	2 (25)	3 (43)		11 (26)
Mechanism of Action				*0.016*	
Cytotoxic Chemotherapy	2 (7)	0 (0)	0 (0)		2 (5)
Targeted Agents	25 (93)	5 (63)	7 (100)		37 (88)
Immune Regulators	0 (0)	3 (38)	0 (0)		3 (7)
**FDA Approval**					
Orphan Drug Designation	1 (4)	0 (0)	0 (0)	*1.000*	1 (2)
Fast Track Designation	7 (26)	1 (13)	2 (29)	*0.763*	10 (24)
Accelerated Approval	2 (7)	4 (50)	2 (29)	*0.012*	8 (19)
Priority Review	20 (74)	5 (63)	6 (86)	*0.689*	31 (74)
Breakthrough Therapy [b]	10 (48)	1 (17)	3 (60)	*0.341*	14 (44)
**Indication Characteristics**					
Indication Approval Type				*0.663*	
Original indication approval	15 (56)	6 (75)	4 (57)		25 (60)
Supplemental indication approval	12 (44)	2 (25)	3 (43)		17 (40)
Treatment Type				*0.000*	
Combination	19 (70)	8 (100)	0 (0)		27 (64)
Monotherapy	8 (30)	0 (0)	7 (100)		15 (36)
Biomarker				*0.082*	
HR positive	12 (44)	0 (0)	1 (14)		13 (31)
HER2 positive	5 (19)	4 (50)	4 (57)		13 (31)
Other	7 (26)	2 (25)	1 (14)		10 (24)
None	3 (11)	2 (25)	1 (14)		6 (14)
Line of Therapy				*0.009*	
First-line	12 (44)	6 (75)	0 (0)		18 (43)
Second-line	13 (48)	1 (13)	4 (57)		18 (43)
≥Third-line	2 (7)	1 (13)	3 (43)		6 (14)
**Pivotal Clinical Trial Characteristics**					
Enrolled patients, median (IQR)	621 (431-726)	629 (393-991)	543 (184-991)	*0.831*	585 (417-752)
Accrual rate, median (IQR) [c]	38 (17-52)	39 (17-65)	23 (15-31)	*0.198*	34 (17-49)
Clinical trial sites, median (IQR)	180 (144-228)	193 (113-228)	169 (72-213)	*0.706*	181 (142-223)
Participating countries, median (IQR)	21 (17-30)	23 (17-35)	15 (8-26)	*0.150*	20 (17-19)
Clinical Trial Phase				*0.154*	
Phase 2	2 (7)	1 (13)	2 (29)		5 (12)
Phase 3	25 (93)	7 (88)	5 (71)		37 (88)
Trial Design				*0.024*	
Single-arm	0 (0)	0 (0)	2 (29)		2 (5)
Randomized-controlled	27 (100)	8 (100)	5 (71)		40 (95)
Type of Blinding				*0.012*	
Open-Label	11 (41)	3 (38)	7 (100)		21 (50)
Double-Blind	16 (59)	5 (63)	0 (0)		21 (50)
Primary endpoint					
Overall Survival	2 (7)	0 (0)	1 (14)	*0.501*	3 (7)
Progression-Free Survival	21 (78)	5 (63)	3 (43)	*0.183*	29 (69)
Tumor Response	0 (0)	2 (25)	2 (29)	*0.012*	4 (10)
Total Concurrent RCTs, No.					
Direct Comparator				*0.003*	
Inactive (Placebo or No Treatment)	20 (74)	7 (88)	0 (0)		27 (68)
Active (Cancer Drug)	7 (26)	1 (13)	5 (100)		13 (33)
Randomization ratio [d]				*0.377*	
Equal	14 (52)	6 (75)	4 (80)		24 (60)
Skewed	13 (48)	2 (25)	1 (20)		16 (40)
Crossover				*0.241*	
Not specified	14 (52)	5 (63)	4 (80)		23 (58)
Allowed	0 (0)	1 (13)	0 (0)		1 (3)
Not allowed	13 (48)	2 (25)	1 (20)		16 (40)
**Total No. of Indications**	27 (64)	8 (19)	7 (17)		42 (100)

Table e2. Stratification by product type: small molecules vs. antibodies vs. antibody-drug conjugates

Notes: This table presents the drug, indication, special FDA designations and review programs, and pivotal clinical trial characteristics for indications with FDA approval for breast cancer. Approvals were compared across product types for small molecules, antibodies, and antibody-drug conjugates.

Abbreviations: FDA, US Food and Drug Administration; HER2, Human epidermal growth factor receptor 2; HR, hormone receptor; IQR, interquartile range; RCT, randomized controlled trial.

[a] P Values calculated based on Fisher's-exact-tests or Kruskal-Wallis-tests.
[b] The comparison of the breakthrough therapy designation only includes indications approved after 2012, given that the program was initiated in 2012.
[c] The accrual rate was calculated as the number of patients enrolled in the clinical trial per month.
[d] Randomization ratios were stratified by the allocation proportion to the treatment and control arm in equal (e.g. 1:1, 1:1:1) and skewed (e.g. 2:1, 3:1, 2:1:1).

No. (%)							
	No. of special FDA designations						Overall
Variables	0	1	2	3	4	*P Value* [a]	
**Drug Characteristics**							
Indication novelty						*0.000*	
Addition-to-indication	8 (100)	3 (30)	0 (0)	0 (0)	0 (0)		11 (26)
Advance-in-indication	0 (0)	4 (40)	13 (72)	2 (50)	1 (50)		19 (45)
First-in-indication	0 (0)	3 (30)	5 (28)	2 (50)	1 (50)		10 (24)
Mechanism of Action						*1.000*	
Cytotoxic Chemotherapy	0 (0)	1 (10)	1 (6)	0 (0)	0 (0)		2 (5)
Targeted Agents	8 (100)	8 (80)	15 (83)	4 (100)	2 (100)		37 (88)
Immune Regulators	0 (0)	1 (10)	2 (11)	0 (0)	0 (0)		3 (7)
**Indication Characteristics**							
Indication Approval Type						*0.129*	
Original indication approval	6 (75)	5 (50)	13 (72)	1 (25)	0 (0)		25 (60)
Supplemental indication approval	2 (25)	5 (50)	5 (28)	3 (75)	2 (100)		15 (36)
Treatment Type						*0.867*	
Combination	4 (50)	7 (70)	12 (67)	3 (75)	1 (50)		27 (64)
Monotherapy	4 (50)	3 (30)	6 (33)	1 (25)	1 (50)		15 (36)
Biomarker						*0.158*	
HR positive	5 (63)	1 (10)	5 (28)	2 (50)	0 (0)		13 (31)
HER2 positive	3 (38)	3 (30)	5 (28)	0 (0)	2 (100)		13 (31)
Other	0 (0)	3 (30)	6 (33)	1 (25)	0 (0)		10 (24)
None	0 (0)	3 (30)	2 (11)	1 (25)	0 (0)		6 (14)
Line of Therapy						*0.565*	
First-line	4 (50)	5 (50)	8 (44)	1 (25)	0 (0)		18 (43)
Second-line	2 (25)	4 (40)	9 (50)	2 (50)	1 (50)		18 (43)
≥Third-line	2 (25)	1 (10)	1 (6)	1 (25)	1 (50)		6 (14)
**Pivotal Clinical Trial**							
Enrolled patients, median (IQR)	1312 (503-3822)	630 (341-808)	541 (417-726)	417 (137-669)	398 (184-612)	*0.227*	585 (417-752)
Accrual rate, median (IQR) [b]	105 (24-229)	31 (16-37)	39 (23-50)	24 (4-42)	20 (16-23)	*0.205*	34 (17-49)
Clinical trial sites, median (IQR)	349 (102-576)	194 (172-204)	186 (158-223)	96 (31-142)	114 (72-155)	*0.044*	181 (142-223)
Participating countries, median (IQR)	33 (15-39)	25 (19-29)	20 (17-29)	16 (7-19)	12 (8-15)	*0.067*	20 (17-19)
Clinical Trial Phase						*0.001*	
Phase 2	0 (0)	0 (0)	1 (6)	5 (12)	0 (0)		5 (12)
Phase 3	8 (100)	10 (100)	17 (94)	37 (88)	100 (0)		37 (88)
Trial Design						*0.017*	
Single-arm	0 (0)	0 (0)	0 (0)	2 (5)	0 (0)		2 (5)
Randomized-controlled	8 (100)	10 (100)	18 (100)	40 (95)	100 (0)		40 (95)
Type of Blinding						*0.925*	
Open-Label	5 (63)	4 (40)	9 (50)	21 (50)	62.5 (0)		21 (50)
Double-Blind	3 (38)	6 (60)	9 (50)	21 (50)	37.5 (0)		21 (50)
Primary endpoint							
Overall Survival	0 (0)	1 (10)	2 (11)	3 (7)	0 (0)	*1.000*	3 (7)
Progression-Free Survival	4 (50)	7 (70)	14 (78)	29 (69)	50 (0)	*0.578*	29 (69)
Tumor Response	0 (0)	1 (10)	1 (6)	4 (10)	0 (0)	*0.180*	4 (10)
Total Concurrent RCTs, No.							
Direct Comparator						*0.684*	
Inactive (Placebo or No Treatment)	4 (50)	7 (70)	12 (67)	27 (66)	50 (0)		27 (66)
Active (Cancer Drug)	4 (50)	3 (30)	6 (33)	14 (34)	50 (0)		14 (34)
Randomization ratio [c]						*0.036*	
Equal	8 (100)	6 (60)	9 (50)	24 (60)	100 (0)		24 (60)
Skewed	0 (0)	4 (40)	9 (50)	16 (40)	0 (0)		16 (40)
Crossover						*0.279*	
Not specified	7 (88)	4 (40)	9 (50)	23 (58)	87.5 (0)		23 (58)
Allowed	0 (0)	1 (10)	0 (0)	1 (3)	0 (0)		1 (3)
Not allowed	1 (13)	5 (50)	9 (50)	16 (40)	12.5 (0)		16 (40)
**Total No. of Indications**	8 (19)	10 (24)	18 (43)	4 (10)	2 (0)		42 (100)

Table e3. Stratification by number of special designations

Notes: This table presents the drug, indication, special FDA designations and review programs, and pivotal clinical trial characteristics for indications with FDA approval for breast cancer. Approvals were compared by the total number of special FDA designations.

Abbreviations: FDA, US Food and Drug Administration.

[a] P Values calculated based on Fisher's-exact-tests or Kruskal-Wallis-tests.

[b] The accrual rate was calculated as the number of patients enrolled in the clinical trial per month.

[c] Randomization ratios were stratified by the allocation proportion to the treatment and control arm in equal (e.g. 1:1, 1:1:1) and skewed (e.g. 2:1, 3:1, 2:1:1).

No. (%)						
	**FDA approvals for breast cancer**					**Overall**
**Variables**	**HR positive**	**HER2 positive**	**Other**	**None**	***P Value*** [a]	
**Drug Characteristics**						
Indication novelty					*0.132*	
Addition-to-indication	5 (38)	5 (38)	0 (0)	1 (17)		11 (26)
Advance-in-indication	7 (54)	5 (38)	6 (60)	2 (33)		20 (48)
First-in-indication	1 (8)	3 (23)	4 (40)	3 (50)		11 (26)
Mechanism of Action					*0.003*	
Cytotoxic Chemotherapy	0 (0)	0 (0)	0 (0)	2 (33)		2 (5)
Targeted Agents	13 (100)	13 (100)	8 (80)	3 (50)		37 (88)
Immune Regulators	0 (0)	0 (0)	2 (20)	1 (17)		3 (7)
**FDA Approval**						
Orphan Drug Designation	0 (0)	1 (8)	0 (0)	0 (0)	*1.000*	1 (2)
Fast Track Designation	3 (23)	5 (38)	1 (10)	1 (17)	*0.501*	10 (24)
Accelerated Approval	0 (0)	3 (23)	3 (30)	2 (33)	*0.123*	8 (19)
Priority Review	8 (62)	8 (62)	10 (100)	5 (83)	*0.086*	31 (74)
Breakthrough Therapy [b]	6 (46)	4 (31)	4 (40)	2 (33)	*0.897*	16 (38)
**Indication Characteristics**						
Indication Approval Type					*0.433*	
Original indication approval	10 (77)	6 (46)	6 (60)	3 (50)		25 (60)
Supplemental indication approval	3 (23)	7 (54)	4 (40)	3 (50)		17 (40)
Treatment Type					*0.727*	
Combination	10 (77)	8 (62)	6 (60)	3 (50)		27 (64)
Monotherapy	3 (23)	5 (38)	4 (40)	3 (50)		15 (36)
Line of Therapy					*0.534*	
First-line	7 (54)	4 (31)	5 (50)	2 (33)		18 (43)
Second-line	5 (38)	6 (46)	5 (50)	2 (33)		18 (43)
≥Third-line	1 (8)	3 (23)	0 (0)	2 (33)		6 (14)
**Pivotal Clinical Trial Characteristics**						
Enrolled patients, median (IQR)	669 (521-726)	612 (417-991)	355 (302-521)	757 (722-1174)	*0.016*	585 (417-752)
Accrual rate, median (IQR) [c]	46 (40-61)	23 (17-41)	20 (13-39)	31 (25-37)	*0.031*	34 (17-49)
Clinical trial sites, median (IQR)	174 (142-223)	204 (161-276)	192 (145-209)	159 (30-199)	*0.598*	181 (142-223)
Participating countries, median (IQR)	22 (19-30	26 (16-29)	18 (17-29)	19 (2-29)	*0.806*	20 (17-19)
Clinical Trial Phase					*0.286*	
Phase 2	0 (0)	3 (23)	1 (10)	1 (17)		5 (12)
Phase 3	13 (100)	10 (77)	9 (90)	5 (83)		37 (88)
Trial Design					*0.502*	
Single-arm	0 (0)	1 (8)	0 (0)	1 (17)		2 (5)
Randomized-controlled	13 (100)	12 (92)	10 (100)	5 (83)		40 (95)
Type of Blinding					*0.376*	
Open-Label	4 (31)	8 (62)	5 (50)	4 (67)		21 (50)
Double-Blind	9 (69)	5 (38)	5 (50)	2 (33)		21 (50)
Primary endpoint						
Overall Survival	0 (0)	1 (8)	0 (0)	2 (33)	*0.059*	3 (7)
Progression-Free Survival	11 (85)	7 (54)	10 (100)	1 (17)	*0.001*	29 (69)
Tumor Response	0 (0)	2 (15)	0 (0)	2 (33)	*0.055*	4 (10)
Total Concurrent RCTs, No.						
Direct Comparator					*0.697*	
Inactive (Placebo or No Treatment)	10 (77)	8 (62)	6 (60)	5 (83)		29 (69)
Active (Cancer Drug)	3 (23)	5 (38)	4 (40)	1 (17)		13 (31)
Randomization ratio [d]					*0.040*	
Equal	6 (46)	11 (92)	4 (40)	3 (60)		24 (60)
Skewed	7 (54)	1 (8)	6 (60)	2 (40)		16 (40)
Crossover					*0.319*	
Not specified	7 (54)	9 (75)	4 (40)	3 (60)		23 (58)
Allowed	0 (0)	1 (8)	0 (0)	0 (0)		1 (3)
Not allowed	6 (46)	2 (17)	6 (60)	2 (40)		16 (40)
**Total No. of Indications**	13 (31)	13 (31)	10 (24)	6 (14)		42 (100)

Table e4. Stratification by biomarkers: HR positive vs. HER2 positive vs. other vs. none

Notes: This table presents the drug, indication, special FDA designations and review programs, and pivotal clinical trial characteristics for indications with FDA approval for breast cancer. Approvals were compared across biomarker status: HR positive vs. HER2 positive vs. other vs. none.

Abbreviations: FDA, US Food and Drug Administration; HER2, Human epidermal growth factor receptor 2; HR, hormone receptor; IQR, interquartile range; RCT, randomized controlled trial.

[a] P Values calculated based on Fisher’s-exact-tests or Kruskal-Wallis-tests.
[b] The comparison of the breakthrough therapy designation only includes indications approved after 2012, given that the program was initiated in 2012.
[c] The accrual rate was calculated as the number of patients enrolled in the clinical trial per month.
[d] Randomization ratios were stratified by the allocation proportion to the treatment and control arm in equal (e.g. 1:1, 1:1:1) and skewed (e.g. 2:1, 3:1, 2:1:1).

No. (%)				
	Treatment setting			Overall
**Variables**	**Metastatic breast cancer**	**Early breast cancer**	***P Value*** [a]	
**Drug Characteristics**				
Indication novelty			*0.049*	
Addition-to-indication	7 (19)	4 (67)		11 (26)
Advance-in-indication	18 (50)	2 (33)		20 (48)
First-in-indication	11 (31)	0 (0)		11 (26)
Mechanism of Action			*0.557*	
Cytotoxic Chemotherapy	2 (6)	0 (0)		2 (5)
Targeted Agents	32 (89)	5 (83)		37 (88)
Immune Regulators	2 (6)	1 (17)		3 (7)
**FDA Approval**				
Orphan Drug Designation	1 (3)	0 (0)	*1.000*	1 (2)
Fast Track Designation	10 (28)	0 (0)	*0.308*	10 (24)
Accelerated Approval	7 (19)	1 (17)	*0.681*	8 (19)
Priority Review	29 (81)	2 (33)	*0.032*	31 (74)
Breakthrough Therapy [b]	12 (46)	2 (33)	*0.672*	16 (50)
**Indication Characteristics**				
Indication Approval Type			*0.374*	
Original indication approval	20 (56)	5 (83)		25 (60)
Supplemental indication approval	16 (44)	1 (17)		17 (40)
Treatment Type			*1.000*	
Combination	23 (64)	4 (67)		27 (64)
Monotherapy	13 (36)	2 (33)		15 (36)
Biomarker			*0.220*	
HR positive	12 (33)	1 (17)		13 (31)
HER2 positive	9 (25)	4 (67)		13 (31)
Other	10 (28)	0 (0)		10 (24)
None	5 (14)	1 (17)		6 (14)
Line of Therapy			*0.587*	
First-line	14 (39)	4 (67)		18 (43)
Second-line	16 (44)	2 (33)		18 (43)
≥Third-line	6 (17)	0 (0)		6 (14)
**Pivotal Clinical Trial Characteristics**				
Enrolled patients, median (IQR)	540 (384-723)	1745 (1174-2840)	*0.007*	585 (417-752)
Accrual rate, median (IQR) [c]	31 (16-42)	85 (46-225)	*0.011*	34 (17-49)
Clinical trial sites, median (IQR)	174 (142-209)	384 (181-549)	*0.057*	181 (142-223)
Participating countries, median (IQR)	19 (16-29)	33 (21-40)	*0.062*	20 (17-19)
Clinical Trial Phase			*0.557*	
Phase 2	4 (11)	1 (17)		5 (12)
Phase 3	32 (89)	5 (83)		37 (88)
Trial Design			*0.732*	
Single-arm	2 (6)	0 (0)		2 (5)
Randomized-controlled	34 (94)	6 (100)		40 (95)
Type of Blinding			*1.000*	
Open-Label	18 (50)	3 (50)		21 (50)
Double-Blind	18 (50)	3 (50)		21 (50)
Primary endpoint				
Overall Survival	3 (8)	0 (0)	*1.000*	3 (7)
Progression-Free Survival	29 (81)	0 (0)	*0.000*	29 (69)
Tumor Response	2 (6)	2 (33)	*0.091*	4 (10)
Total Concurrent RCTs, No.				
Direct Comparator			*0.697*	
Inactive (Placebo or No Treatment)	22 (65)	5 (83)		27 (68)
Active (Cancer Drug)	12 (35)	1 (17)		13 (33)
Randomization ratio [d]			*0.373*	
Equal	19 (56)	5 (83)		24 (60)
Skewed	15 (44)	1 (17)		16 (40)
Crossover			*1.000*	
Not specified	19 (56)	4 (67)		23 (58)
Allowed	1 (3)	0 (0)		1 (3)
Not allowed	14 (41)	2 (33)		16 (40)
**Total No. of Indications**	36 (86)	6 (14)		42 (100)

Table e5. Stratification by treatment setting: early vs. metastatic breast cancer

Notes: This table presents the drug, indication, special FDA designations and review programs, and pivotal clinical trial characteristics for indications with FDA approval for breast cancer. Approvals were compared by treatment setting comparing early and metastatic breast cancer.

Abbreviations: FDA, US Food and Drug Administration; HER2, Human epidermal growth factor receptor 2; HR, hormone receptor; IQR, interquartile range; RCT, randomized controlled trial.

[a] P Values calculated based on Fisher's-exact-tests or Kruskal-Wallis-tests.
[b] The comparison of the breakthrough therapy designation only includes indications approved after 2012, given that the program was initiated in 2012.
[c] The accrual rate was calculated as the number of patients enrolled in the clinical trial per month.
[d] Randomization ratios were stratified by the allocation proportion to the treatment and control arm in equal (e.g. 1:1, 1:1:1) and skewed (e.g. 2:1, 3:1, 2:1:1).

	Median	(95% CI)	*P Value*
Line of therapy			
First-line	16013	(13911-17610)	
Advanced-line	15373	(12909-20911)	*0.970*
Improvement in QoL			
No	16013	(13284-17624)	
Yes	16243	(16146-17610)	*0.319*
Improvement in OS			
No	16013	(13284-16934)	
Yes	16079	(12699-19260)	*0.891*
Improvement in QoL or OS			
No	16013	(13284-17624)	
Yes	16146	(12909-17610)	*0.729*
ESMO-MCBS			
Low-value	16243	(13284-20911)	
High-value	16013	(12489-16243)	*0.299*
**Total**	**16013**	**(13097-17617)**	

Table e6. Median monthly price of breast cancer drugs

Notes: All prices in 2023 USD.

Abbreviations: ESMO, European Society for Medical Oncology; MCBS, Magnitude of Clinical Benefit Scale; OS overall survival; QoL, quality of life.
